# Frequency and spectrum of *PIK3CA* somatic mutations in breast cancer

**DOI:** 10.1186/s13058-020-01284-9

**Published:** 2020-05-13

**Authors:** Olga Martínez-Sáez, Nuria Chic, Tomás Pascual, Barbara Adamo, Maria Vidal, Blanca González-Farré, Esther Sanfeliu, Francesco Schettini, Benedetta Conte, Fara Brasó-Maristany, Adela Rodríguez, Débora Martínez, Patricia Galván, Ana Belén Rodríguez, Antonio Martinez, Montserrat Muñoz, Aleix Prat

**Affiliations:** 1 Department of Medical Oncology, Hospital Clinic of Barcelona, Villarroel 170, 08035 Barcelona, Spain; 2 Translational Genomics and Targeted Therapies in Solid Tumors, Institut D’Investigacions Biomèdiques August Pi i Sunyer (IDIBAPS), Villarroel 170, 08035 Barcelona, Spain; 3 SOLTI Breast Cancer Research Group, Barcelona, Spain; 4 Pathology Department, Hospital Clinic of Barcelona, Villarroel 170, Barcelona, 08035 Spain; 5 University of Napoles Federico II, Napoles, Italy

**Keywords:** Breast cancer, *PIK3CA*, Mutations, Alpelisib, Companion diagnostic, Hotspot mutations, Therascreen, ctDNA

## Abstract

**Purpose:**

The therascreen *PIK3CA* mutation assay and the alpha-specific PI3K inhibitor alpelisib are FDA-approved for identifying and treating patients with advanced *PIK3CA-*mutated (*PIK3CA*mut) breast cancer (BC). However, it is currently unknown to what extend this assay detects most *PIK3CA* mutations in BC. This information is critical as patients and clinicians are using this and other genomic assays to indicate alpelisib.

**Methods:**

Data from 6338 patients with BC was explored across 10 publicly available studies. The primary objective was to evaluate the proportion and distribution of *PIK3CA* mutations in BC. Secondary objectives were (1) to evaluate in silico the spectrum of *PIK3CA* mutations in BC that would be captured by the therascreen panel; (2) to evaluate the proportion and distribution of *PIK3CA* mutations in hormone receptor-positive/HER2-negative (HR+/HER2−), HER2+, and triple-negative BC (TNBC); and (3) to explore the identification of *PIK3CA* mutations in a cohort of 48 HR+/HER2− advanced BC patients by the Guardant B360 circulating tumor DNA (ctDNA) assay.

**Results:**

Patients with *PIK3CA*mut tumors represented 35.7% (2261/6338). Five *PIK3CA* mutations comprised 73% of all *PIK3CA* mutations: H1047R (35%), E545K (17%), E542K (11%), N345K (6%), and H1047L (4%). Therascreen gene list would capture 72% of all *PIK3CA* mutations and 80% of patients with a known *PIK3CA*mut BC. Among patients with double *PIK3CA*mut tumors (12% of all *PIK3CA*mut), the therascreen panel would capture 78% as harboring 1 single *PIK3CA* mutation, 17% as *PIK3CA*mut undetected, and 5% as *PIK3CA* double-mut. *PIK3CA* mutation rates were lower in TNBC (16%) compared to HR+/HER2 (42%) and HER2+ (31%) BC; however, the distribution of the 4 main *PIK3CA* mutations across subtypes was similar. Finally, 28% of *PIK3CA* mutations identified in ctDNA in 48 patients with advanced HR+/HER2− BC were not part of the therascreen panel.

**Conclusion:**

*PIK3CA* mutations in BC are heterogenous and ~ 20% of patients with a known *PIK3CA* mutation, and 95% with a known double *PIK3CA*mut tumor, would not be captured by the therascreen panel. Finally, the clinical utility of *PIK3CA* mutations not present in the therascreen companion diagnostic assay or identified by other sequencing-based assays needs further investigation.

## Introduction

Activating mutations in the *PIK3CA* are found in approximately 30–40% of patients with cancer and induce hyperactivation of the alpha isoform (p110α) of the phosphatidylinositol 3-kinase (PI3K) [1–3]. In patients with HR+/HER2− BC, mTOR/mTOR pathway has been associated with endocrine therapy resistance [4]. In addition, the role of this pathway is becoming increasingly important in HER2+ and TNBC [5–7]. Thus, inhibition of PI3K in *PIK3CA-*mutated BC has been a major focus in the last decade [3].

Alpelisib is an orally bioavailable, small-molecule, α-specific PI3K inhibitor that inhibits p110α approximately 50 times as strongly as other isoforms [8]. Following successful preclinical and phase 1 data [4, 9], the SOLAR-1 phase III randomized trial evaluated the efficacy of alpelisib plus fulvestrant in 572 patients with HR+/HER2− advanced BC who had received prior endocrine therapy [10]. A clinically relevant treatment benefit was only observed in the cohort of patients with *PIK3CA*mut disease. In May 2019, the FDA approved alpelisib for the treatment of patients with advanced *PIK3CA*mut HR+/HER2− BC.

Together with alpelisib, the FDA also approved the companion diagnostic therascreen® *PIK3CA* test (QIAGEN Manchester, Ltd.) used in SOLAR-1 to select patients who had *PIK3CA* mutations in tumor tissue specimens and/or in circulating tumor DNA (ctDNA) isolated from plasma specimens [11]. Therascreen *PIK3CA* detects 11 *PIK3CA* hotspot mutations, mostly found in exons 9 and 20 [11]. In SOLAR-1, the type of *PIK3CA* mutation did not seem to impact the main results [10].

In this context, patients and physicians might choose not to use the therascreen *PIK3CA* test and use other available tests, which provide a more comprehensive mutational analysis of *PIK3CA* as well as other genes. This might lead to the clinical situation where *PIK3CA* mutations not detected by the therascreen *PIK3CA* assay, and thus not evaluated in SOLAR-1, are used to indicate alpelisib. To define the potential frequency of this clinical situation, here we aimed to evaluate the distribution of *PIK3CA* mutations in BC in relation to the therascreen *PIK3CA* panel.

## Methods

### Datasets

**Fig. 1:**
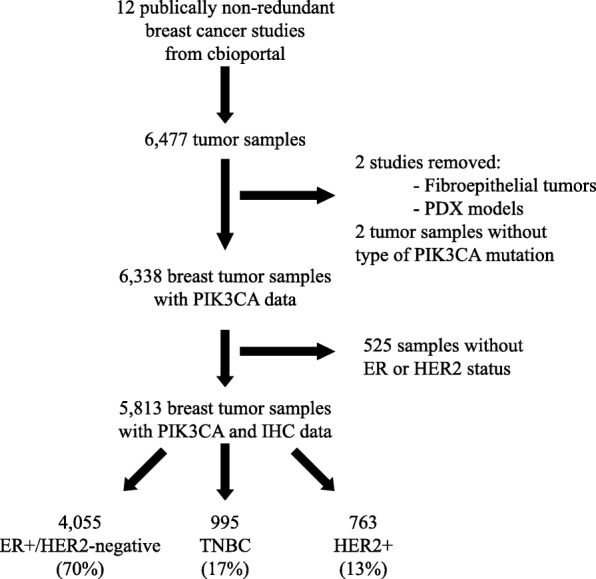
The CONSORT diagram

All non-overlapping publicly available breast datasets (i.e., 12 studies and 6477 samples) (https://www.mbcproject.org/data-release [1, 12–21]) with *PIK3CA* mutational status were interrogated from cBio Cancer Genomics Portal (http://cbioportal.org) [22] (Fig. 1 and Additional file 1). Among them, 2 studies focused on 117 patient-derived xenografts [16] and 22 fibroepithelial lesions of the breast [17] were removed. The remaining combined dataset included 6338 invasive tumor samples of which 5535 (87.3%) originated from the METABRIC (*n* = 2509), the Memorial Sloan-Kettering (*n* = 1918), and The Cancer Genome Atlas (*n* = 1108) datasets. All studies analyzed performed targeted or whole exome sequencing (Table A1 Additional file 1). Only single nucleotide variants, insertions, or short deletions in *PIK3CA* were analyzed.

### Therascreen *PIK3CA* mutation assay

The therascreen® *PIK3CA* RGQ PCR Kit is a real-time qualitative PCR test for the detection of 11 mutations in *PIK3CA* gene (exon 7: C420R; exon 9: E542K, E545A, E545D, E545G, E545K, Q546E, and Q546R; and exon 20: H1047L, H1047R, and H1047Y) using genomic DNA extracted from formalin-fixed, paraffin-embedded breast tumor tissue or ctDNA from plasma derived from K2EDTA anticoagulated peripheral whole blood taken from patients with BC [11].

### Immunohistochemistry (IHC)-based subtypes

IHC data was available from 5813 patients (92%). Tumors were classified into the 3 main clinically relevant subtypes: (1) HR+/HER2−, (2) HER2+, and (3) TNBC. Tumors identified as progesterone receptor positive and HER2− were considered HR+ regardless of estrogen receptor (ER) status. Tumors identified as ER-negative and HER2− were considered TNBC when progesterone receptor status was not available.

### Distribution of *PIK3CA* mutations in plasma

Frozen plasma samples from 48 patients with advanced HR+/HER2− BC were obtained before initiating a CDK4/6 inhibitor and endocrine therapy. Plasma samples were sent to Guardant Health (California, USA), and the 74-gene standardized NGS-based assay, which includes all 21 exons from the *PIK3CA* gene, was performed.

### Study end points

Primary objective was to evaluate the proportion and distribution of *PIK3CA* mutations in BC. Secondary objectives were (1) to evaluate in silico the spectrum of *PIK3CA* mutations in BC that would be captured by the therascreen panel; (2) to evaluate the proportion and distribution of *PIK3CA* mutations in HR+/HER2−, HER2+, and TNBC; and (3) to explore the identification of *PIK3CA* mutations in HR+/HER2− advanced BC by the Guardant B360 ctDNA assay.

### Statistical analyses

Patient and tumor characteristics were analyzed using descriptive statistics.

## Results

### Distribution of *PIK3CA* mutations in BC

**Fig. 2:**
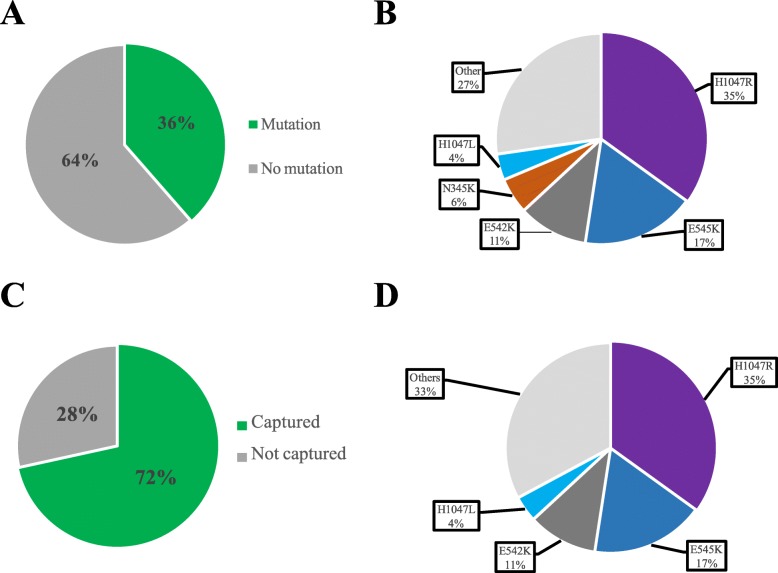
Proportion of *PIK3CA* mutations in BC in relation to the mutations detected by therascreen. **a** Proportion of patients with *PIK3CA* mutations in the combined dataset. **b** Distribution of the various types of *PIK3CA* mutations in the combined dataset. **c** Proportion of *PIK3CA* mutations detected by the therascreen assay. **d** Distribution of the various types of *PIK3CA* mutations detected by the therascreen assay in the combined dataset

In the combined dataset, 36% of patients had *PIK3CA*mut tumors (Fig. 2a). From a total of 2560 *PIK3CA* mutations, 205 *PIK3CA* mutations were unique. The most frequent *PIK3CA* mutations (i.e., frequency ≥ 4% of all *PIK3CA*mut tumors) were found in exons 4, 9, and 20: H1047R (35%), E545K (17%), E542K (11%), N345K (6%), and H1047L (4%) (Table 1, Fig. 2b). These 5 mutations comprised 73% of all *PIK3CA* mutations identified in the combined dataset.

**Table 1:** The 20 most frequent *PIK3CA* mutations in BC

Type of *PIK3CA* mutation	Exon	Oncogenic by OncoKB^24^	Level of evidence to predict alpelisib benefit	Detected by therascreen	Number of mutations found in the combined dataset	Mutation frequency (%)
H1047R	20	Yes	1	Yes	895	35.0
E545K	9	Yes	1	Yes	447	17.5
E542K	9	Yes	1	Yes	274	10.7
N345K	4	Yes	Yes (preclinical only)	No	142	5.5
H1047L	20	Yes	1	Yes	103	4.0
E726K	13	Inconclusive. Probably oncogenic	Unknown	No	65	2.5
C420R	7	Yes	1	Yes	48	1.9
Q546R	9	Yes	1	Yes	27	1.1
G118D	1	Yes	Unknown	No	26	1.0
E453K	7	Yes	Unknown	No	22	0.9
Q546K	1	Yes	Yes (preclinical only)	No	21	0.8
G1049R	20	Yes	Yes (preclinical only)	No	19	0.7
M1043I	20	Yes	Unknown	No	19	0.7
K111E	1	Yes	Unknown	No	16	0.6
E81K	1	Inconclusive. Probably oncogenic	Unknown	No	15	0.6
E545A	9	Yes	1	Yes	13	0.5
E545G	9	Yes	1	Yes	13	0.5
N1044K	20	Yes	Unknown	No	12	0.5
E110del	1	Yes	Unknown	No	11	0.4
Q546P	9	Yes	Unknown	No	10	0.4

### *PIK3CA* mutations captured by the therascreen panel

**Fig. 3:**
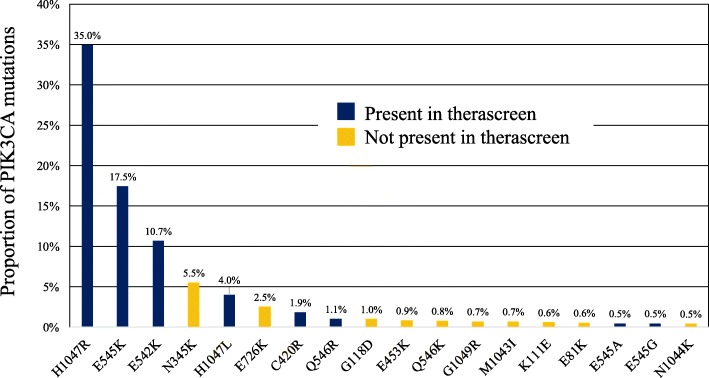
Proportion of the 18 most frequent *PIK3CA* mutations in *PIK3CA*mut BC in the combined dataset

In the combined dataset, the proportion of *PIK3CA* mutations included in the therascreen panel was 72% (Fig. 2c). The most frequent types of *PIK3CA* mutation (i.e., frequency ≥ 4% of all *PIK3CA*mut tumors) included in the therascreen panel were H1047R (35%), E545K (17%), E542K (11%), and H1047L (4%) (Fig. 2d). These 4 mutations comprised 67% of all *PIK3CA* mutations detected in the dataset. Of note, N345K mutation in exon 4, which represents 6% of all tumor samples with a *PIK3CA* mutation, is not part of the therascreen panel (Fig. 3). Although the clinical utility of non-therascreen-detected *PIK3CA* mutations is currently unknown, the N345K lies within the C2 PI3K-type domain of the protein and confers a gain of function on PI3K, as does C420R (a tested mutation by therascreen assay) [23]. Moreover, N345K mutation has shown increased sensitivity to PI3K pathway inhibition in preclinical models [24]. Interestingly, Q546E *PIK3CA* mutation included in the therascreen panel was not found in the combined dataset.

### Detection of multiple *PIK3CA* mutations in a tumor sample

**Fig. 4:**
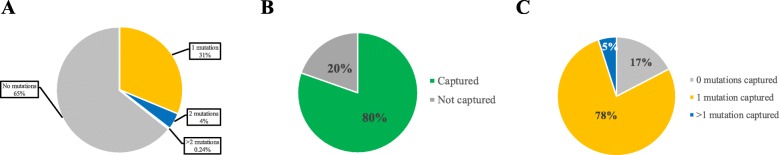
Proportion of patients with *PIK3CA* mutations in BC in relation to the mutations detected by therascreen. **a** Proportion of patients with one, two, or three or more *PIK3CA* mutations in BC in the combined dataset. **b** Proportion of patients with a single *PIK3CA* mutation detected *PIK3CA*mut by the therascreen assay. **c** Proportion of patients with two or more *PIK3CA* mutations detected by the therascreen assay as either *PIK3CA* mutation “not detected,” single *PIK3CA* mutation or as harboring 2 or more *PIK3CA* mutations

Among 2261 patients with *PIK3CA*mut tumors, 1979 (87.5%) had 1 single mutation, 267 (11.8%) had 2 mutations, and 15 (0.7%) had 3 or more mutations. Overall, patients with tumors harboring double *PIK3CA* mutations represented 4% of all BC (Fig. 4a). Among patients with 1 single *PIK3CA* mutation, 80% would have mutations represented in the therascreen mutational panel (Fig. 4b). Among patients with 2 or more *PIK3CA* mutations, 78% would have 1 mutation represented in the therascreen panel; 17%, no mutation represented in the therascreen panel; and 5%, 2 or more mutations represented in the therascreen panel (Fig. 4c).

### *PIK3CA* mutational distribution according to subtypes in the BC dataset

**Fig. 5:**
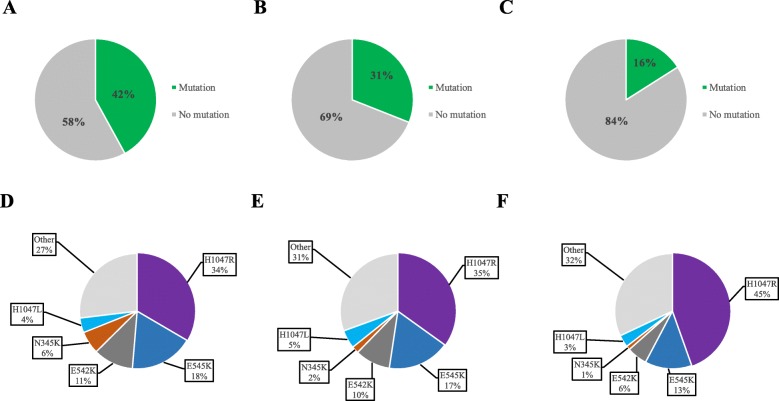
Proportion of *PIK3CA* mutations across the BC subtypes. **a** Proportion of *PIK3CA* mutations in HR+/HER2-negative BC. **b** Proportion of *PIK3CA* mutations in HER2+ BC. **c** Proportion of *PIK3CA* mutations in TNBC. **d** Distribution of the various types of *PIK3CA* mutations in *PIK3CA*mut HR+/HER2-negative BC. **e** Distribution of the various types of *PIK3CA* mutations in *PIK3CA*mut HER2+ BC. **f** Distribution of the various types of *PIK3CA* mutations in *PIK3CA*mut TNBC

Among 5813 patients with IHC data, 4055 (70%) had HR+/HER2− disease, 995 had TNBC (17%), and 763 (13%) had HER2+ disease. *PIK3CA* mutations were less frequent in TNBC (16%) than in HR+/HER2− (42%) or HER2+ disease (31%) (Fig. 5a–c). However, the distribution of *PIK3CA* mutations was similar across subtypes (Fig. 5d–f). Seventy-one percent of mutations in HR+/HER2− BC, 75% in HER2+ BC, and 72% in TNBC would be represented in the therascreen panel.

### Distribution of *PIK3CA* mutations in plasma ctDNA

Therascreen assay is approved for detecting *PIK3CA* mutations in ctDNA from plasma samples [25]. To evaluate the distribution of *PIK3CA* mutations in ctDNA using a highly sensitive assay that sequences all 21 *PIK3CA* exons, we tested plasma samples from 48 consecutive patients with metastatic HR+/HER2− BC from the Hospital Clinic of Barcelona using the Guardant B360 standardized assay [26]. All patients had recurred or progressed to prior lines and were about to initiate a CDK4/6 inhibitor and endocrine therapy. A *PIK3CA* mutation was detected in 17 patients (37%), and 1 patient (6%) had double *PIK3CA* mutation. The spectrum of *PIK3CA* mutations was similar to the one found in the previous in silico population analysis (Table A2 Additional file 1). More importantly, 5 patients (28%) had *PIK3CA* mutations not represented in the therascreen mutational panel.

## Discussion

*PIK3CA* mutations have recently reached level 1 evidence for predicting benefit from alpelisib, an alpha-specific PI3K inhibitor, in combination with fulvestrant in patients with advanced HR+/HER2− BC previously treated with endocrine therapy [10]. In addition, several trials are now evaluating alpelisib and other alpha-specific PI3K inhibitors in other BC subtypes harboring *PIK3CA* mutations [27]. Thus, there is a need to better understand the heterogeneity of the mutational landscape of *PIK3CA* and, at the same time, relate this heterogeneity with the recently introduced therascreen *PIK3CA* companion diagnostic assay approved to indicate alpelisib.

To address this topic, we performed a comprehensive evaluation of the distribution of *PIK3CA* mutations in BC and made the following observations. First, although *PIK3CA* mutations are highly heterogeneous, 5 mutations (H1047R, E545K, E542K, N345K, and H1047L) represented ~ 70% of all known types of *PIK3CA* mutations in the dataset. Second, the therascreen *PIK3CA* mutational panel would represent 72% of all the known *PIK3CA* mutations and 80% of all patients with a known *PIK3CA* mutation. Third, 83% of patients with 2 or more *PIK3CA* known mutations would have mutations found in the therascreen panel; however, in 78% of the cases, only 1 single *PIK3CA* mutation would be represented in the therascreen assay. Finally, the proportion of *PIK3CA* mutations differed by BC subtype with HR+/HER2− disease having the highest proportion, followed by HER2+ disease and TNBC. Although less frequent in the HER2+ and TNBC, the proportion is not negligible and several studies, including pivotal or registrational clinical trials, are focusing on these two populations [5–7]. To our knowledge, this is the first report to perform a comprehensive analysis of *PIK3CA* mutations in BC and to relate these findings with the type of mutations captured by the therascreen *PIK3CA* assay across the three main subtypes of BC.

The SOLAR-1 phase III trial that led to the approval of alpelisib used the therascreen *PIK3CA* 11-mutation assay in tumor tissue to identify *PIK3CA* mutations [10]. From a total of 1173 patients tested for *PIK3CA* mutation status that had interpretable results, 341 (29%) patients had *PIK3CA*mut disease [10], a proportion which is very similar (28%) to our predicted results if the assay would have been performed in our combined dataset. More importantly, mutations in exon 9 versus exon 20 predicted similarly the degree of benefit to alpelisib in SOLAR-1 [10]. Thus, based on these results, the FDA approved the use of this assay in tumor and plasma samples as a companion diagnostic to indicate alpelisib. The approval of therascreen in plasma samples is based on a subanalysis of the SOLAR-1 trial which showed that *PIK3CA* mutations identified in plasma samples were also associated with treatment benefit [28].

Our results have important considerations for patients and physicians. In certain parts of the world, determination of *PIK3CA* status is commonplace using various types of sequencing-based assays. Some of these widely used assays such as Foundation One CDx or Guardant360 cover most or all exons of the *PIK3CA* gene. Thus, it is highly likely that mutations which are not part of the therascreen *PIK3CA* 11-mutation assay will be identified with other assays and treatment decisions will be made. In other parts of the world that have not yet implemented somatic genetic testing in BC, the fact that the therascreen panel misses ~ 20–30% of patients with known *PIK3CA* mutations might be a reason to choose more comprehensive *PIK3CA* panels.

Critical questions raised by our results are if patients with *PIK3CA* mutations which are not part of the therascreen panel, or hotspot and non-hotspot *PIK3CA* mutations identified using sequencing-based assays with higher sensitivities than therascreen, will benefit from alpelisib. For example, mutation N345K represented 5.5% of all *PIK3CA* mutations in the analyzed dataset and is not captured by the therascreen assay. This mutation was the fourth most frequent *PIK3CA* mutation in the BC dataset, and COSMIC [29] and OncoKB [30] datasets consider it pathogenic (score 0.95) and oncogenic. Moreover, N345K confers a gain of function and it has shown to increase sensitivity to PI3K inhibitors in preclinical models [23, 24]. A similar situation exists for the sixth most frequently observed *PIK3CA* mutation, E726K, although OncoKB [30] states that there is conflicting and/or weak data describing the oncogenic function of this mutation, it has been shown that as a single mutation it is weakly activating but as a double mutation (with E545K or H1047R) it is synergistically activating [31]. It is important to notice that the vast majority of E726K mutations are found precisely as double mutants in BC [31]. On the other hand, some less frequent mutations, as G1049R, have demonstrated strong driver activity in a mutation assessment platform. G1049R exhibited activity levels similar to the E542K variant with 20-fold higher frequency [24]. Thus, better functional characterization of these and other non-hotspot *PIK3CA* mutations together with clinical evidence that predict benefit to alpelisib and other alpha-specific PI3K inhibitors is now of uttermost importance. At the end of the day, each type of *PIK3CA* mutation should be considered a biomarker by itself.

Another interesting observation is that ~ 4% of all BC, or ~ 12% of all patients with *PIK3CA*mut BC, have double *PIK3CA* mutations. Preclinically, double compound *PIK3CA* mutations result in increased PI3K activity and downstream signaling compared to single hotspot mutants in nontransformed cells and in HR+ BC cells [31]. More importantly, these compound mutations seem to predict for increased sensitivity to PI3K alpha-specific inhibitors compared to single hotspot mutants in both preclinical models and also in selected patients with BC treated in early phase 1 trials [31]. According to our results, the therascreen panel would not capture well double *PIK3CA* mutations since only 5% of patients known to harbor 2 or more *PIK3CA* mutations would have mutations represented in the therascreen panel. Thus, if double mutations are confirmed to be a biomarker of ultra-high sensitivity to alpelisib, the therascreen assay might not be ideal for this purpose.

Our study has limitations worth noting. First, we did not evaluate the actual analytical concordance of the therascreen assay versus other sequencing assays. In other words, we assumed that the results of the combined dataset using various sequencing-based strategies was the gold standard and that the therascreen assay would identify 100% of all the *PIK3CA*-wild-type tumors as “no *PIK3CA* mutation detected” and 100% of all the *PIK3CA*mut tumors in the combined dataset as “*PIK3CA*mut” if the type of mutation was on the therascreen mutation panel. However, the differences in the sensitive and specificity of the various sequencing assays will affect the concordance rates among them [32, 33]. According to the FDA therascreen *PIK3CA* assay specification sheet, the overall percent agreement between the therascreen assay and an NGS-based assay in SOLAR-1 was 94.7%. Second, the next-generation sequencing assays and the methods used across the 10 studies evaluated in our study are highly heterogeneous and most are not standardized. Third, the analyzed datasets were mostly from primary tumor samples and acquisition of new *PIK3CA* mutations has been described in the metastatic setting in 8–10% of the cases [34]. Whether the frequency and spectrum of *PIK3CA* mutations would change if metastatic-only samples had been analyzed is currently unknown.

## Conclusion

*PIK3CA* somatic mutations in BC are highly heterogenous, and the currently validated therascreen companion diagnostic test, which covers 11 hotspot mutations, might not capture up to 20% of patients with *PIK3CA* mutations. Thus, there is an urgent need to better understand if patients with *PIK3CA* mutations not detected by the therascreen assay, and predicted to be oncogenic and activating, can benefit from alpelisib or other PI3K inhibitors. Better functional characterization of these and other non-hotspot *PIK3CA* mutations together with further clinical studies in tumor and plasma samples from SOLAR-1 and other studies will help to better determine the population of patients who benefit from alpelisib or other alpha-specific PI3K inhibitors.

## Supplementary information

**Additional file 1: Table A1.** Main features of the 10 publicly available studies analyzed. **Table A2.***PIK3CA* mutations found in plasma ctDNA (Guardant B360 assay) in a cohort of patients from Hospital Clinic of Barcelona.

## Data Availability

The datasets analyzed during the current study are available in http://cbioportal.org.
