# Supplementary material for: Frequency and spectrum of PIK3CA somatic mutations in breast cancer
Source: Breast Cancer Res. 2020 May 13;22:45. doi: 10.1186/s13058-020-01284-9 (PMC7222307; doi:10.1186/s13058-020-01284-9)
Supplement: Supplementary file 1 — Additional file 1: Table A1. Main features of the 10 publicly available studies analyzed. Table A2.PIK3CA mutations found in plasma ctDNA (Guardant B360 assay) in a cohort of patients from Hospital Clinic of Barcelona. [file 13058_2020_1284_MOESM1_ESM.docx]

**Table A1**. Main features of the 10 publicly available studies analyzed.

|  | **Authors** | **Year** | **Link** | **Title** | **Patients** | **Samples** | **Sequencing technique** |
| --- | --- | --- | --- | --- | --- | --- | --- |
| **Study1** | Razavi et al. | 2019 | <https://www.ncbi.nlm.nih.gov/pubmed/30205045> | The Genomic Landscape of Endocrine-Resistant Advanced Breast Cancers | 1756 | 1918 | MSK-IMPACT, a hybridization capture-based next-generation sequencing assay, which analyzes all protein-coding exons of between 341 and 468 cancer-associated genes |
| **Study2** | Pereira et al. | 2016 | <https://www.ncbi.nlm.nih.gov/pubmed/27161491> | The somatic mutation profiles of 2,433 breast cancers refines their genomic and transcriptomic landscapes | 2509 | 2509 | Targeted exome sequencing of 173 genes |
| **Study3** | MSK, unpublished | 2019 | MSK, unpublished | Targeted Sequencing of buparlisib + letrozole and alpelisib + letrozole-treated metastatic ER+ unmatched breast tumors | 68 | 70 | MSK-IMPAT, Targeted exome sequencing |
| **Study4** | Shah et al. | 2012 | <https://www.ncbi.nlm.nih.gov/pubmed/22495314> | The clonal and mutational evolution spectrum of primary triple-negative breast cancers | 65 | 65 | Genome/exome sequence |
| **Study5** | Banerji et al. | 2012 | <https://www.ncbi.nlm.nih.gov/pubmed/22722202> | Sequence analysis of mutations and translocations across breast cancer subtypes | 103 | 103 | Whole-exome sequencing |
| **Study6** | Stephens et al. | 2012 | <https://www.ncbi.nlm.nih.gov/pubmed/22722201> | The landscape of cancer genes and mutational processes in breast cancer | 100 | 100 | Exome sequencing |
| **Study7** | TCGA | 2012 | <https://www.ncbi.nlm.nih.gov/pubmed/23000897> | Comprehensive molecular portraits of human breast tumours | 1101 | 1108 | Whole exome sequencing |
| **Study8** | Lefebvre et al. | 2016 | <https://www.ncbi.nlm.nih.gov/pubmed/28027327> | Mutational Profile of Metastatic Breast Cancers: A Retrospective Analysis | 216 | 216 | Whole-exome sequencing |
| **Study9** | The Metastatic Breast Cancer Project | 2018 | unpublished | The Metastatic Breast Cancer Project | 237 | 237 | Whole Exome Sequencing |
| **Study10** | Martelotto et al. | 2015 | <https://www.ncbi.nlm.nih.gov/pubmed/26095796> | Genomic landscape of adenoid cystic carcinoma of the breast | 12 | 12 | Whole Exome Sequencing |

**Table A2.** *PIK3CA* mutations found in plasma ctDNA (Guardant B360 assay) in a cohort of patients from Hospital Clinic of Barcelona.

| Type of *PIK3CA m*utation | Mutation frequency, n (%) | Captured by the therascreen panel |
| --- | --- | --- |
| E545K | 6 (35%) | Yes |
| H1047R | 4 (24%) | Yes |
| H1047L | 1 (6%) | Yes |
| C420R | 1 (6%) | Yes |
| E453_P458del | 1 (6%) | No |
| E542K | 1 (6%) | Yes |
| E726K* | 1 (6%) | No |
| G364R | 1 (6%) | No |
| H1047I | 1 (6%) | No |
| P104_V105del | 1 (6%) | No |

*Doble mutation
